# Expanding the biomass derived chemical space

**DOI:** 10.1039/c7sc00936d

**Published:** 2017-04-24

**Authors:** Nicolas Brun, Peter Hesemann, Davide Esposito

**Affiliations:** a Max-Planck-Institute of Colloids and Interfaces , 14424 Potsdam , Germany . Email: davide.esposito@mpikg.mpg.de; b Institut Charles Gerhardt , UMR 5253 CNRS – Université de Montpellier – ENSCM , Place Eugène Bataillon , 34095 Montpellier cédex 05 , France

## Abstract

Biorefinery aims at the conversion of biomass and renewable feedstocks into fuels and platform chemicals, in analogy to conventional oil refinery. In the past years, the scientific community has defined a number of primary building blocks that can be obtained by direct biomass decomposition. However, the large potential of this “renewable chemical space” to contribute to the generation of value added bio-active compounds and materials still remains unexplored. In general, biomass derived building blocks feature a diverse range of chemical functionalities. In order to be integrated into value-added compounds, they require additional functionalization and/or covalent modification thereby generating secondary building blocks. The latter can be thus regarded as functional components of bio-active molecules or materials and represent an expansion of the renewable chemical space. This perspective highlights the most recent developments and opportunities for the synthesis of secondary biomass derived building blocks and their application to the preparation of value added products.

## Abstract 2

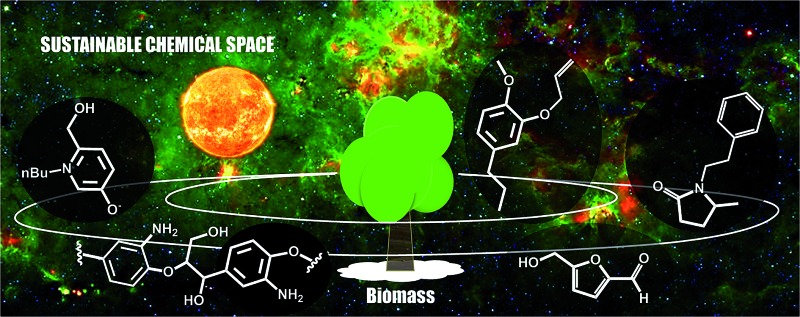
The derivatization and covalent modification of biomass derived platform chemicals expand the biomass derived chemical spaces allowing for the preparation of new bioactive molecules and materials.

## 1. Introduction

In the past years, societal concerns related to anthropogenic changes of the ecosystem have become an important part of the political agenda.^1^ Sustainability and ecological performance are today important criteria for green process engineering. In this context, the concept of biorefinery emerged as a valuable and challenging strategy to reduce the environmental impact of modern chemical industry and the related production chains. Biorefinery deals with the conversion and valorisation of biogenic renewable feedstock, *e.g.* biomass. It is based on the sustainable processing of biomass into a spectrum of marketable products and energy.^2,3^ The analogy with a conventional oil refinery has been often evoked to define the scope and the nature of biorefinery. Thus, fuels and a relatively limited number of platform chemicals have been identified as the target products of the valorisation of renewable raw materials. However, the differences with conventional refinery of fossil resources are quite remarkable. In particular, the respective feedstocks feature different physicochemical characteristics; oil is usually homogeneous and liquid (it may contain a gaseous fraction) with a low oxygen content, while biomass is essentially an oxygen rich heterogeneous solid. This implies differences in the chemical strategies required for their processing. Moreover, also their availability is quite different. Fossil resources are finite in nature and usually localized in specific geological formations, which include conventional reservoirs as well as oil shales and sands. Biomass in turn is renewable and variably spread over the planet, with a yearly abundance that is limited by the efficiency of photosynthesis. In addition, biomass is the feedstock for different sectors, including the food, agriculture, forestry and energy sectors. This highlights the need for systems integration research, in order to define the impact of biomass utilization across interconnected areas and avoid negative environmental impacts.^4^ Today, for instance, there is a relatively strong consensus that a “sustainable biorefinery” should not prioritize on the generation of fuels, which are required in very high volumes, but rather on the productions of specialty and platform chemicals. In fact, the total replacement of fossil fuels with biofuels on a global scale would require the reconversion of all available agriculture land,^5^ with obvious sustainability and societal problems.

The production of chemicals and materials from biomass traces back before the beginning of the industrial era. Among the first examples of materials that could be directly obtained from nature are natural rubbers and cellulose. Since the industrial revolution, scientists from different disciplines have contributed to the development of efficient strategies to deconstruct biomass in order to enable economy of scale. In this regard, the extraction of cellulose from trees for the production of paper has been one of the benchmark processes for the valorization of lignocellulosic biomass.^6^ Beside cellulose extraction however, a number of strategies have been developed in the last decades that allow for the deconstruction of biomass and the production of an array of different chemicals.^7^ We have previously classified such processes as degradative (DD) and non-degradative (NDD) deconstruction processes, referring to the possibility of decomposing biomass into small organic compounds (*e.g.* furans, levulinic acid, small aromatics *etc.*) or into its non-degraded biopolymer constituents (*e.g.* cellulose, hemicellulose and lignin) respectively.^8^ Particularly interesting is the case of degradative processes, which target C1 to C6 compounds. In this context, since biomass is highly oxygenated, processing strategies are generally based on hydrolysis, dehydration, hydrodeoxygenation, hydrogenolysis or a combination thereof, in order to remove or modify functional groups, whereas oxidations steps are usually employed to restore specific chemical functionalities.^9–12^ The products of DD and NDD processes can be regarded as the primary biomass derived building blocks. Many publications have highlighted the “platform” nature of such compounds, surveying the direct transformations they could undergo in order to generate a number of different commodities.^13^ However, if a market already exists for such primary biomass derived building blocks is the subject of debate. If the chemical is a compound that can also be obtained on the basis of conventional fossil precursors, then the economic viability of the process plays a significant role. On the other side, if the building block is novel and features chemical functionalities and connectivity that are different than the ones found in conventional platform chemicals, it becomes a candidate for novel applications, with the potential to open up a new chemical space.^14^ A good example in this direction is the case of lactic acid that has resulted in the development of polylactide as biodegradable plastic, which is not in competition with conventional oil based materials.^15^ On a more general level however, the challenge remains to identify potential applications for such biomass derived primary building blocks: this represents a crucial criterion to justify the biorefinery scheme that is required for their production.

**Scheme 1:**
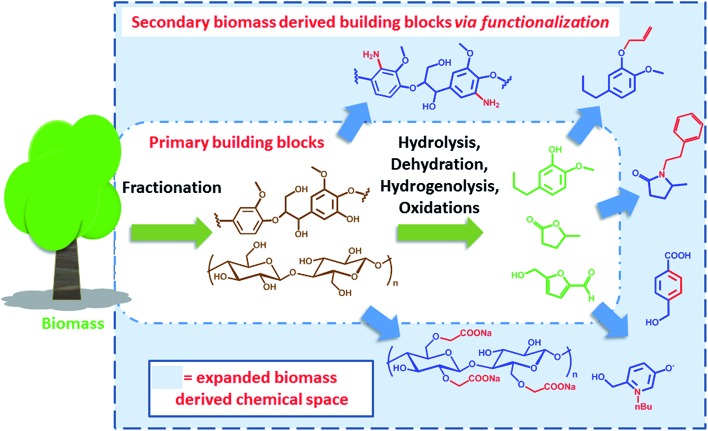
Schematic representation of the expanded biomass derived chemical space.

Clearly, some of the primary building blocks of biomass degradation can find an almost direct application. Besides paper, for instance, cellulose has been extensively used for the preparation of hydrogels^16^ or employed as support for nanoparticles.^17^ Other building blocks like cyrene,^18^ 2-methyl tetrahydrofuran (2-MTHF) or γ-valerolactone have found direct applications due to their interesting solvent properties and have been even employed for biomass processing^19^ and fractionation.^20^ However, for many applications such building blocks cannot be utilized “as synthesized”. In order to become part of value added products, *e.g.* bio-active compounds or new materials, biomass derived primary building blocks need to be functionalized or covalently linked with additional compounds in order to generate secondary building blocks featuring additional functionalities and properties. Such molecules represent an expansion of the chemical space that can be accessed on the basis of biomass derived molecules (Scheme 1).

This perspective will highlight some recent methods and opportunities for the synthesis of secondary biomass derived building blocks and their subsequent transformation into value added products. In particular, we will discuss those processes that are based on the functionalization and covalent modification of biomass derived molecules. The overall idea is to provide the reader with an overview of the currently explored sustainable chemical space. Examples that have the potential to reach commercial application will be included. In fact, identifying value added products that can be prepared on the basis of secondary biomass derived building blocks will be of crucial importance for the success of biorefinery and for its technology transfer to industry. For simplicity, lignocellulose derived compounds will be primarily discussed here. However, similar considerations can be applied to compounds derived from a different renewable feedstock and selected examples will also be recalled. The modification/functionalization of cellulose, which represents a more established field, was not included in the present discussion and we redirect the reader to specialized literature.^21,22^ Examples involving hydrolysis, dehydration, hydrodeoxygenation, hydrogenolysis or oxidations of biomass derived molecules will not be discussed here, as they fall into the repertoire of transformation for the preparation of “primary building blocks”.

## 2. Biomass derived chemical space for the synthesis of bio-active compounds

Compared to classical synthetic chemistry based on petrochemical intermediates, biorefinery follows a different philosophy. Traditionally, derivatization methods of oil-derived platform chemicals involve oxidation steps to introduce chemical functionalities. In contrast, biorefinery processes have been targeting the removal of functionalities in order to simplify the complexity of biomolecules. However, the real challenge is performing such processes in a selective way, preserving those existing chemical functionalities that can be exploited for synthetic purposes.^23^ In this context, biomass offers many challenging opportunities to access bioactive fine chemicals, thanks to the relatively complex constitution of bio-sourced building blocks. As mentioned in the introduction, many primary biomass derived building blocks can be considered as starting materials for the synthesis of functional compounds and materials. This section deals with recent examples based on the synthesis of secondary biomass derived building blocks that feature interesting biological properties. Such compounds can also be regarded as intermediates for the synthesis of pharmaceutically relevant compounds. In particular, we will focus on examples related to the functionalization and derivatization of levulinic acid, γ-valerolactone, and finally furfural and hydroxymethylfurfural. Moreover, sustainable chemical approaches (heterogeneous catalysis, catalysis with cheap and non-toxic catalysts such as iron or bio-sourced catalysts/organocatalysts) are discussed preferentially.

### 2.1 Levulinic acid and related esters

**Scheme 2:**
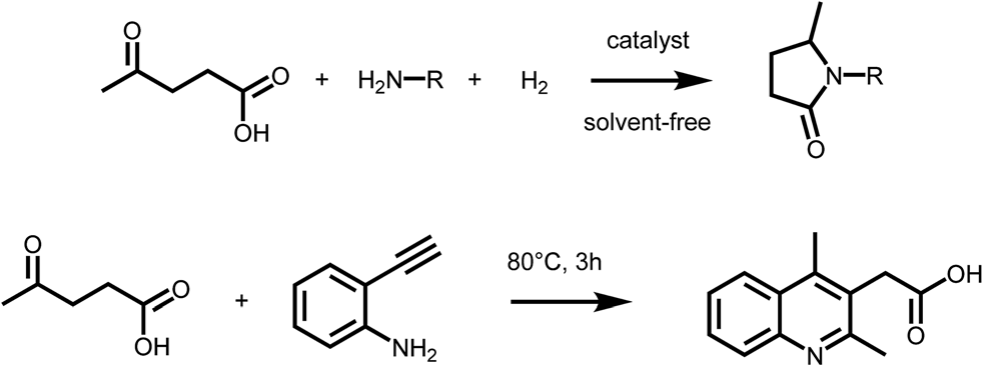
(Upper) Synthesis of pyrrolidones *via* reductive amination of LA; (lower) formation of quinolines form LA and 2-alkynylanilines.

Levulinic acid (LA) is generally obtained *via* the acid catalyzed solvolysis of sugars such as glucose or fructose. During the reaction, hydroxymethylfurfural (HMF) is formed as the key intermediate, which is further converted to levulinic acid and formic acid.^8^ Levulinic acid has high importance as a precursor for many primary biomass derived building blocks, including the biofuels γ-valerolactone (GVL), 2-MTHF or levulinic esters.^9^ It is therefore a key intermediate in biorefinery.^24^ However, LA is a very interesting building block also for the organic synthesis of fine chemicals, besides its conventional use as protecting group. The formation of substituted pyrrolidones *via* reductive amination reactions has received particular attention in the recent past (Scheme 2 – upper). It has been reported that LA can be converted into a wide range of *N*-substituted 5-methyl-pyrrolidones by reductive amination with different amines followed by a cyclisation step. The synthetic approach, which relies on the use of LA, a primary amine and hydrogen, is highly versatile and efficient, thus leading to a large variety of compounds with potential applications as industrial solvents, surfactants, and complexing agents. However, pyrrolidones are a key component of many bio active compounds, as for instance nootropic drugs such as racetams, which can be obtained by proper selection of the amine.^25^ To date, several heterogeneous catalytic systems have been employed for such process, including platinum, ruthenium or iron–nickel alloys based catalysts.^25–27^ Interestingly, the formation of pyrrolidones was also shown to occur under catalyst- and solvent-free conditions,^28^ or using formic acid as a renewable hydrogen source^29^ pointing to the possibility of decreasing the environmental impact of this approach. A very similar synthetic strategy was recently employed to access substituted quinolines. Interestingly, the reaction of LA with 2-ethynylaniline afforded 2-(2,4-dimethyl-quinolin-3-yl) acetic acid^26^ (Scheme 2 – lower). It was shown that a series of substituted quinolines can be obtained using different 2-alkynylanilines. Noteworthy, besides levulinic acid, levulinic esters are also suitable starting materials for reductive amination–cyclisation sequences,^30^ showing in some cases higher reactivity.

Nitrogen containing heterocycles are not the only target of LA derivatization. For instance, Lin *et al.* reported a new method for the thionation of LA using Lawesson's reagent. In this way, different thiophenones as well as 5-methylthiophene-2-thiol could be obtained in good yields even using crude LA prepared by cellulose hydrolysis.^31^ While the direct application of such secondary biomass derived building blocks for the preparation of bioactive compounds was not shown, thiophenes are commonly used in drugs and agrochemicals.^32^

Besides its role in the synthesis of heterocycles, additional aspects of levulinic acid's reactivity render this compound interesting for biomedical applications. Thanks to its keto and acid groups, levulinic acid readily undergoes imine formation or acylation reactions. Both kinds of linkages are susceptible to cleavage under physiological conditions, pointing to possible exploitation of LA for the preparation of cleavable linker systems. For example, LA was recently used in the synthesis of a linker for a new class of anti-tumoral conjugate vaccines based on α-galactosylceramide (α-GalCer) derived prodrugs.^33^ Furthermore, LA has been used as a bifunctional spacer for the synthesis of a series of substituted hydrazones whose copper(ii) complexes were identified as potent antitubercular agents.^34^

### 2.2 γ-Valerolactone (GVL)

**Scheme 3:**
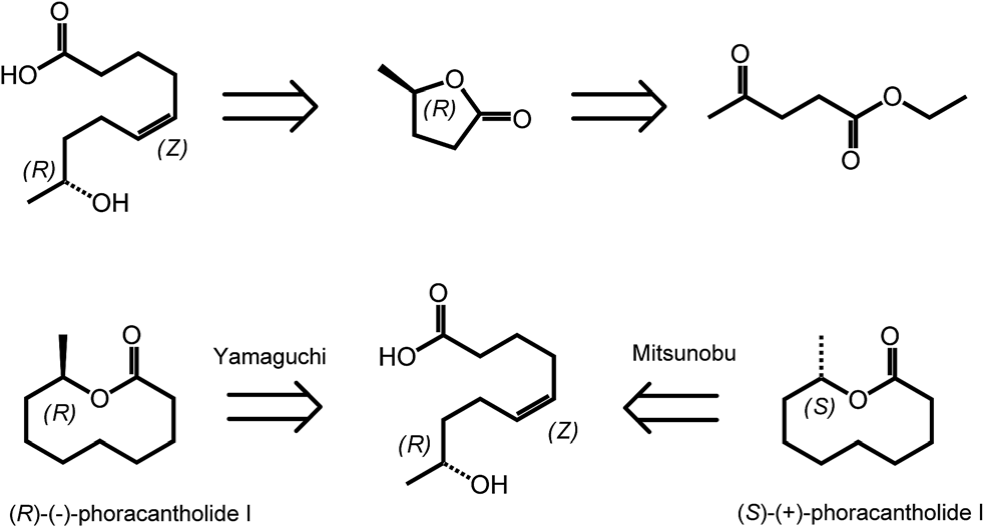
Retrosynthesis of (*R*)-(–)-phoracantholide I and (*S*)-(+)-phoracantholide I from (*R*)-(+)-γ-valerolactone. Adapted from ref. 38.

γ-Valerolactone (GVL) is the cyclic five membered ester obtained from levulinic acid *via* two step sequences involving reduction of the keto group followed by cyclization.^35^ As mentioned in the introduction, GVL has a great potential to be used as biofuel or biosolvent.^20,36^ However, γ-valerolactone has also a particular position in the area of biomass derived synthetic chemistry, as it exists in two enantiomeric forms, (*R*)-(+)-γ-valerolactone and (*S*)-(–)-γ-valerolactone. The availability of valerolactone in its enantiomerically pure form opens large opportunities for the synthesis of chiral biologically active molecules, as lactones are important structural entities in pheromones, polyketides and prostaglandins. Optically pure γ-valerolactone can be obtained from ethyl levulinate *via* enzymatic asymmetric reduction, followed by lactonization.^37^ Very recently, the synthesis of (*R*)-(–)-phoracantholide I and (*S*)-(+)-phoracantholide I, two pheromones found in frogs of the family of mantillidae, has been achieved from (*R*)-(+)-γ-valerolactone in high yields (Scheme 3).^38^ Also chemical strategies have been developed for the preparation of enantiopure-GVL. For instance, levulinic acid could be converted with 82% enantioselectivity into (*S*)-GVL in the presence of a SEGPHOS ligand-modified ruthenium catalyst.^39^ In this paper, the authors also describe different bio-active molecules which can be synthesized employing such a chiral building block.

It is worth mentioning that, beyond the preparation of bio-active molecules, the functionalization of GVL can open up opportunities also in the field of bio-based materials. For example, the methylenation of GVL at its α position affords an interesting methacrylate analogue that has been employed for the preparation of sustainable latexes.^40^ The latter material features very interesting thermal properties compare to traditional acrylate polymers.^41^

### 2.3 Furans: furfural and hydroxymethyl furfural (HMF)

Acid catalyzed dehydration of monosaccharides can be used to access furfural and hydroxymethyl furfural (HMF).^8^ Both compounds have potential as platform chemicals, in particular in the area of biofuels.^42^ Interestingly, HMF is also the key intermediate in the synthesis of levulinic acid (*vide supra*) and γ-valerolactone. However, due to its pattern of functional groups, HMF represents a very interesting candidate for the preparation of high value bio-active compounds. Reductive amination reactions represent a straightforward possibility to expand the chemical space accessible by furans introducing nitrogen containing functionalities. Due to their large versatility, reductive aminations of furfurals are at present intensively studied. As an example, a method was recently proposed that uses ammonia as the amine source and hydrogen gas in the presence of a Rh/Al_2_O_3_ catalyst.^43^ Interestingly, similar reactions can also be promoted electrochemically with commercial metal electrodes under environmentally benign conditions using water as hydrogen source.^44^ This approach may further decrease the cost and environmental concerns currently associated with conventional reductive amination reactions.

**Scheme 4:**
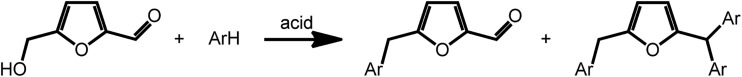
Arylation of HMF. Adapted from ref. 45.

Arylation reactions represent an additional possibility for the derivatization of furfural and HMF. As recently shown, these reactions can afford a large variety of valuable mono-, di- or triarylated intermediates. Superelectrophilic activation using either triflic acid or acidic H-USY zeolites enables arylation reactions and affords a series of substituted furans (Scheme 4). Whereas reaction of HMF with benzene exclusively afforded the phenylmethyl furfural and kept the aldehyde group of furfural intact, similar reactions with more activated arenes such as toluene, xylenes, mesitylene and anisol afforded higher substituted Friedel Crafts products (Scheme 4).^45^ The formed arylated furans may be used to access bioactive compounds such as antibiotics.^46^

**Scheme 5:**
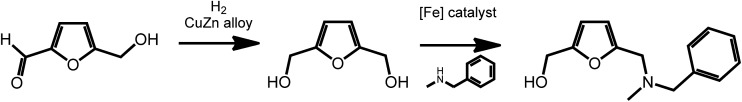
Synthesis of 2-furfuryl-dialkylamines from HMF. Adapted from ref. 49.

2,5-Furan-dimethanol (FDM), an intermediate accessible from HMF,^47,48^ is an interesting starting material for the formation of pharmaceutically active molecules. As recently shown by Barta *et al.*, FDM can readily be obtained *via* reduction procedures involving a noble-metal-free copper–zinc nanoalloy,^47^ and can further be converted to 2-furfuryl-dialkylamines *via* an iron-catalyzed direct alkylation reaction (Scheme 5).^49^ 2-Furfuryl-dialkylamines represent a class of pharmaceutically relevant intermediates. For example, some 2-furfuryl-dialkylamines show antimuscarinic properties. This work shows the easy access of bioactive compounds from renewable platform chemicals using sustainable catalytic processes.

**Scheme 6:**
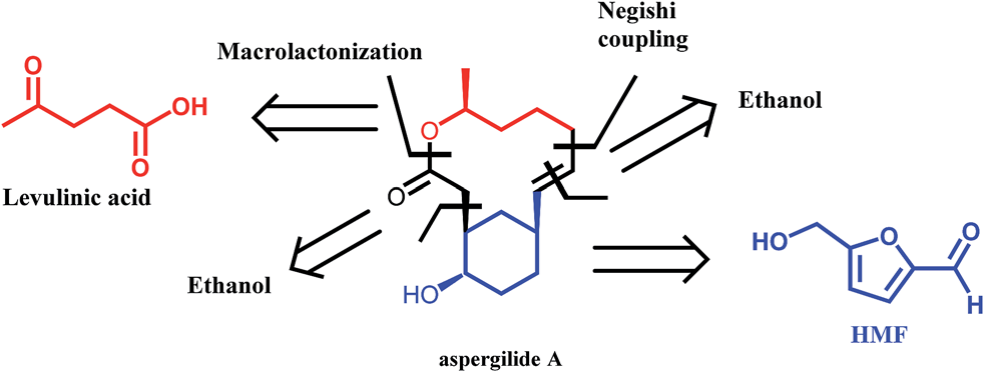
Retrosynthetic approach to aspergilide A. Adapted from ref. 50.

A very interesting synthetic strategy for the preparation of biologically active compounds using exclusively biomass derived platform molecules has recently been reported by Koh *et al.* ^50^ The authors described the syntheses of aspergilide A, B and C, three potent cytotoxic compounds, in which the main synthons for the backbone of the natural products are derived from HMF and levulinic acid (Scheme 6). The multistep synthetic strategy includes among others the use of Noyori's asymmetric transfer hydrogenation, Achmatowicz rearrangement as well as an enzymatic kinetic resolution. This example strongly highlights the potential of biomass derived platform molecules for the synthesis of high value and complex biologically active products.

**Scheme 7:**
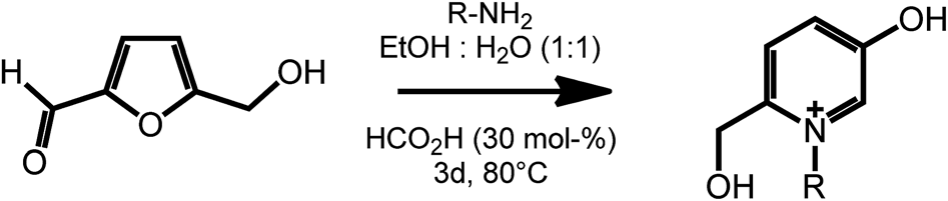
Formation of substituted pyridinium salts by ring expansion of HMF. Adapted from ref. 51.

Another useful feature of HMF is the ability to undergo ring expansion upon formation of an imine with a primary amine. In this way, highly functionalized pyridinium salts can be obtained smoothly under metal free conditions using organocatalyzed transformations (Scheme 7).^51^ This synthetic strategy is of particular interest as pyridinium species are often bioactive compounds, and are present in a high number of natural products. For example, a member of this class of pyridinium salts obtained by reaction of HMF and l-alanine has been identified as a taste enhancer.^52^ Kirchhecker *et al.* ^53^ recently reported a strategy for the preparation of a library of structural analogues of such taste enhancer by reacting furfural with different amino acids. Interestingly, the obtained pyridinium salts could be employed as the starting material for the preparation of pyridinium based ionic liquids *via* a novel hydrothermal decarboxylation method. This example showcases the importance of complementing different sustainable chemical spaces with each other: in particular, combining furfural (from lignocellulose) with amino acids (from proteins) expands the range of accessible functionalities and leads to value added products on a fully renewable basis.

## 3. Biomass derived secondary aromatic building blocks *via* Diels–Alder chemistry

**Scheme 8:**
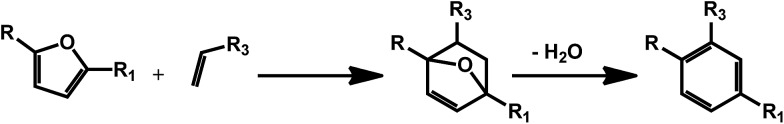
General scheme for the preparation of substituted benzenes using the Diels–Alder/dehydration approach with carbohydrates derived furans.

As mentioned in the previous section, furans obtained by dehydration reactions of carbohydrates offer an interesting set of functionalities and may undergo a variety of interesting transformations. In this regard, cycloaddition reactions deserve a special attention as furans have proven as an optimal substrate for this class of reactions since the early developments.^54^ HMF derivatives have been recently suggested for the preparation of bio-derived aromatics *via* combination of a Diels–Alder reaction and a dehydration step (Scheme 8). This kind of approach can afford a variety of different secondary building blocks and expand the aromatic chemical space accessible on the basis of bio based compounds, depending on the substitution pattern of the furan and the corresponding dienophile. The synthesis of terephthalic acid (TPA) using this approach has received a great deal of attention. The latter compound is a very important commodity for the preparation of polyethylene terephthalate (PET). Although terephthalic acid has been claimed as potential endocrine disruptor and alternatives are currently under investigation, bio-based and partially bio-based routes to conventional plastic materials are expected to receive considerable attention in the next years.^55^ The standard route to TPA involves the oxidation of *p*-xylene. Therefore one of the first efforts has been directed to the synthesis of this compound using biomolecules. In this case, the HMF derivative dimethylfuran^8^ is used in combination with ethylene as the dienophile. This way of generating the partially bio-based commodity is interesting in light of the shale gas revolution,^56^ which can provide ethylene at very competitive prices. However, ethylene could soon become available on the basis of renewable resources, for example *via* ethanol deoxygenation.^57^ Different types of zeolites have been identified as very promising catalysts for the synthesis of TPA. For instance, microporous zeolite were shown to favor at the same time the cycloaddition, by confinement of the substrate within the micropores of the materials, and the catalyzed dehydration step by means of Brønsted acid sites.^58^ Attention has initially been paid to commercially available zeolites; however, novel catalyst preparations are being investigated, in order to improve the efficiency of the process. For example, a novel beta zeolite with a nanosponge-like morphology has recently been prepared showing improved activity.^59^ Also silica–alumina aerogel catalysts have been recently introduced and the authors reported that the yield of *p*-xylene was highly dependent on the silica alumina ratio in the composite material.^60^ Besides xylene, the Diels–Alder/dehydration approach has been also exploited for the preparation of toluene using 2-methylfuran as the diene.^61,62^ The latter can be obtained by hydrolysis/dehydration of hemicellulose.

Targeting xylene as intermediate for the synthesis of TPA implies the reduction of HMF to dimethylfuran followed by a re-oxidation. Therefore, from a sustainability stand point, the use of hydrogen should be taken into consideration. The development of direct routes based on HMF or related oxidized derivatives would be an interesting alternative. Davis *et al.* have recently explored this approach,^63,64^ evaluating the reaction of high pressure ethylene gas with different oxidized derivatives of HMF in autoclaves. Considering the fact that the presence of carboxyl (–CO_2_H) groups on the furan ring results in a very electron-poor and deactivated diene, ether and ester derivatives of 5-(hydroxymethyl)furoic acid were also tested. Zr-Beta was found to be one of the most active catalyst, affording methyl 4-(methoxymethyl)benzene carboxylate with 81% selectivity at 26% diene conversion over 6 h. The possibility of generating TPA on the basis of biomass derived furans is particularly interesting considering the fact that also ethylene glycol, the comonomer required for the synthesis of PET, can be produced nowadays on the basis of renewables. Such bio-based PET precursors are perfect drop-in replacements for the production of an established commodity, as recently discussed by Zhang *et al.* ^65^ As mentioned above, however, PET has been subjected to criticisms due to the potential danger associated with human exposure to terephthalic acid.^66^ In this regard, the dicarboxylic acid derivative of furan (in particular 2,5-furandicarboxylic acid; FDCA) has already proved as promising candidates for the generation of a bio-sourced analogue of PET, the so called PEF^67^ (Polyethylene Furanoate), a material that features very interesting gas permeation properties,^68^ and has received considerable attention in the private sector (see for instance the YXY technology developed by Avantium^69^). Besides terephthalic acids, additional substituted benzene molecules have been targeted using the cycloaddition route. For instance, the group of Lobo has proposed the synthesis of benzoic acids combining furans and acrylates.^70^ Benzoic acid represents a platform chemical that can be utilized for the production of Nylon-6, polycarbonates, epoxy resins and phenolic resins on the basis of existing technologies. Moreover, acrylates, which feature an optimal reactivity for Diels–Alder reaction with furans, can be nowadays prepared from biomass,^71^ pointing to the possibility of obtaining benzoic acid derivatives on a fully renewable basis. One of the limitation associated with the Diels–Alder/aromatization approach based on furans is the relatively high tendency of the adducts to undergo a retro Diels–Alder, especially at the high temperatures required for the aromatization step. For this reason, a novel strategy was recently proposed by Thiyagarajan *et al.* that introduced an intermediate Pd/C catalysed hydrogenation step for the reduction of the cycloaddition product, to effectively block retro Diels–Alder activity.^72^ Thus, conventional zeolites were applied for the aromatization step, as showcased during the synthesis of different substituted phthalic anhydrides.

The simplicity of Diels–Alder reactions represents a very great opportunity to expand the chemical space accessible on the basis of renewable furans. Particularly relevant to the field of green chemistry will be those processes that rely also on renewable dienophiles, as recently exemplified by Hoye *et al.* in an investigation of Diels–Alder process between itaconic acid and furans.^73^ Here, the substrate variability can be quite broad, leading to the synthesis of compounds with increasing values also as synthetic intermediates in organic chemistry.

## 4. From raw to functionalized lignin

**Scheme 9:**
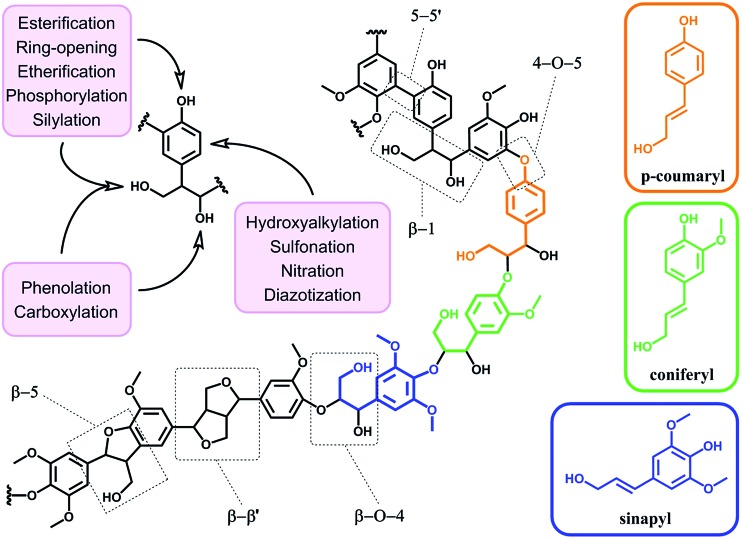
Schematic representation of the structure of lignin with the three main monomer units and prevalent chemical linkages (central structure). Corresponding monolignols – *p*-coumaryl, coniferyl and sinapyl alcohols (right) – and common chemical linkages (dashed boxes). Main reactions reported in literature for the functionalization of lignin (upper left).

In terms of possibility to expand the bio-based aromatic chemical space, lignin is definitely a very attractive target within lignocellulosic biomass feedstocks. This amorphous biopolymer can be found in the cell walls of vascular plants together with hemicellulose and cellulose. It is considered as the second most abundant biopolymer on earth, just after cellulose, and features a complex structure derived from three main monolignols – *i.e. p*-coumaryl, coniferyl and sinapyl alcohols – yielding three main monomer units interlinked through ether or carbon–carbon bonds (Scheme 9).^74^ The ratio of each unit and the linkages between them differ significantly depending on plant species, which makes the exact chemical structure of lignin highly variable and hardly determinable in detail. At a commercial scale, lignin is mainly produced as a by-product of the pulp and paper industry, however it remains largely unutilized, except as low quality combustible.^6^ Beyond abundance and low-cost however, lignin presents many advantageous properties, among which stiffness, high carbon content, antioxidant and antibacterial activities and high thermal stability. For this reason, raw lignins have been suggested as potential precursors for the preparation of bio-based polymeric materials. For example, they have been explored in blends and composites as well as in thermosetting materials,^75^ as antioxidant additives, UV protection agents, flame retardants and reinforcement fillers. In addition, the grafting of polymer chains onto lignin for the preparation of composites has been widely reported.^76^ For a more exhaustive description of this specific research area, we redirect the readers to topical reviews.^74,76^ Here, we will focus on examples related to the chemical modification of lignin *via* functionalization. These approaches provide lignin with peculiar physicochemical properties, making it suitable for a broader range of applications and expanding the biomass derived aromatic chemical space.

**Scheme 10:**
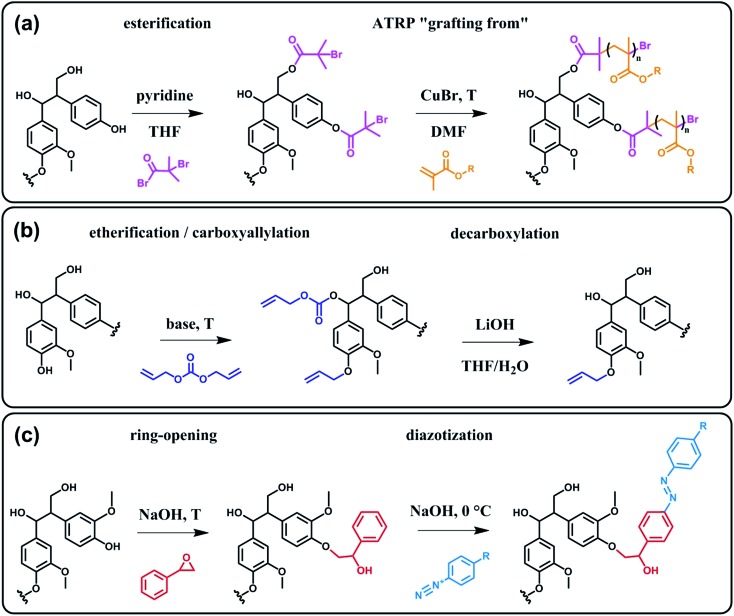
Chemical modification of lignin *via* hydroxyl groups. (a) Esterification with α-bromoisobutyryl bromide to generate lignin-based macroinitiators for the subsequent ATRP of methacrylate monomers. Adapted from ref. 78. (b) Allylation with diallyl carbonate and subsequent decarboxylation of allylated aliphatic hydroxyl groups. Adapted from ref. 80. (c) Benzylation with styrene oxide and subsequent reaction with aryldiazonium cation to form azobenzene compounds. Adapted from ref. 81.

Beyond a complex and heterogeneous connectivity (Scheme 9), lignin features an interesting pattern of functionalities, which is a key target for chemical modifications.^74^ Raw lignin mostly displays methoxy and hydroxyl groups – both aliphatic and phenolic – and may feature a few aldehyde functionalities within their side chains. At this stage, it is important to keep in mind that most of the lignins employed are produced and extracted from chemical pulping processes yielding so-called technical lignins.^74^ Depending on the extraction process, additional minor functionalities can be introduced such as aliphatic thiols and sulfonate groups, as found in kraft and lignosulfonate lignins respectively. Moreover, molecular weight, thermal stability and solubility of lignins are also greatly affected by the extraction/fragmentation method.^77^ For this reason, the full characterization of the targeted lignin is crucial for further chemical modifications. Amongst the different functional groups, phenolic and aliphatic hydroxyl groups are quite abundant and highly reactive. In principle, the functionalization of lignin *via* hydroxyl groups allows developing generic strategies that can be applied independently of the botanical source or the pulping process. Reactions with hydroxyl groups of lignins include esterification^78,79^ (Scheme 10a), etherification^80^ (Scheme 10b), ring-opening reactions^81^ (*e.g.* with epoxides or lactide) (Scheme 10c) and phenolation.^82^ In particular, esterification has been widely employed to incorporate anchorage points for the subsequent grafting of polymer chains. For instance, recent studies proposed the atom transfer radical polymerization (ATRP) of methacrylate monomers from lignin-based macroinitiators (“Grafting from” method; Scheme 10a) for the preparation of gene delivery vectors,^83^ CO_2_ responsive nanoparticles^78^ or even self-healing hydrogels.^84^ Esterification was also used to graft alkyne functionalities. Click reaction between alkyne-modified lignin and azide-terminated polymer was then proposed (“Grafting to” method) for the preparation of composites with self-healing properties.^79^ Besides esterification, etherification represents a further interesting approach. Meier *et al.* ^80^ reported on the allylation of organosolv technical lignin with diallyl carbonate (Scheme 10b). The authors demonstrated the selective etherification of phenols along with the carboxyallylation of aliphatic hydroxyl groups. The accessibility and reactivity of the as-grafted allyl groups was further assessed, opening the way to additional functionalization through metathesis reactions. Moreover, the selective decarboxylation of aliphatic allyl carbonate groups was demonstrated, offering a versatile strategy for further modification or polymerization. In another study, stimuli-responsive lignin was synthesized through benzylation with styrene oxide and subsequent reaction of the exposed benzene groups with aryldiazonium cations (Scheme 10c).^81^

Covalent modifications of aromatic rings *via* aromatic substitutions are other possible solutions to functionalize lignin. Recently, diazotization was performed without prior benzylation directly onto oxidized lignin residues – after alkaline hydrogen peroxide pretreatment.^85,86^ Interestingly, after pyrolysis, nitrogen-doped carbons were obtained and employed as electrode materials for lithium ion batteries.^86^ Diazotization is not the sole aromatic substitution possible to functionalize lignin (Scheme 9). In the same vein, nitration was proposed by Fellinger *et al.* to prepare lignin-derived nitrogen-doped carbon materials.^87^ Such materials showed promising performances as non-noble metal electrocatalysts towards the oxygen reduction reaction. Aromatic sulfonation of lignin with sulfuric acid was also reported by Budarin *et al.* ^88^ The as-synthesized sulfonated lignin particles were tested as heterogeneous acid catalysts in the esterification of levulinic acid. Hydroxyalkylation with formaldehyde has been also widely employed, in particular for the grafting of polyethylenimine through the Mannich reaction.^89^

**Scheme 11:**
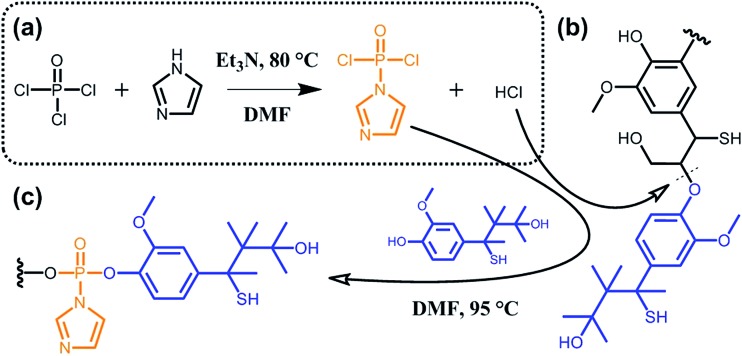
Phosphorylation of kraft lignin. (a) *In situ* synthesis of (imidazol-1-yl)phosphonic dichloride from phosphoryl chloride and imidazole and generation of hydrogen chloride as a side product. (b) Hydrolysis of ether bonds (typically β-*O*-4 bonds) of kraft lignin induced by hydrogen chloride. (c) Phosphorylation of kraft lignin residues. Adapted from ref. 90.

A major issue when aiming at grafting new functionalities to lignin lies in the poor accessibility of the targeted sites due to high molecular weight and considerable steric hindrance. To overcome this limitation, some groups suggested the functionalization of partially deconstructed lignin. Taking the idea one step further, other groups proposed one-pot approaches where depolymerization and chemical modification are concomitant. The overall philosophy herein is to assist deconstruction of lignocellulosic material *via* covalent modifications, thus generating functional secondary building blocks. For instance, Zhu *et al.* ^90^ reported on the phosphorylation of kraft lignin for the preparation of new lubricant additives. As shown in Scheme 11, the authors exploited hydrogen chloride, which is generated *in situ* during the synthesis of (imidazol-1-yl)phosphonic dichloride, to hydrolyze ether bonds (typically β-*O*-4 bonds; Scheme 9). At the same time, the (imidazol-1-yl)phosphonic dichloride acts as a phosphorylating agent for the obtained lignin oligomers. As long as the reaction between (imidazol-1-yl)phosphonic dichloride and lignin residues occurs, hydrogen chloride is generated and the cleavage of ether bonds progresses. Besides the incorporation of new functionalities and the decrease in molecular weight, the solubility of such functional lignin in polar solvents could be significantly improved.^90^

**Scheme 12:**
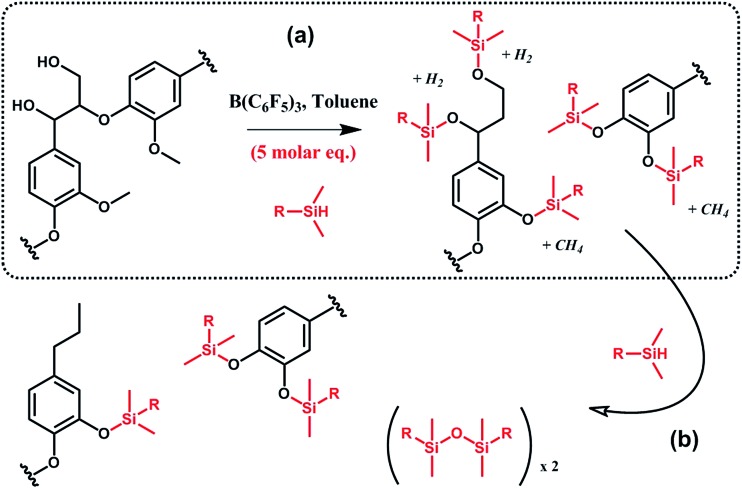
Silylation and reductive degradation of lignin with hydrosilanes in the presence of tris(pentafluorophenyl)borane, B(C_6_F_5_)_3_. (a) First, hydroxyl and methoxy groups react with hydrosilanes to yield aliphatic and aromatic silyl ethers. Gaseous by-products, *i.e.* methane and hydrogen, are formed. (b) Then, providing the reaction medium with an excess of hydrosilanes, aliphatic silyl ethers are fully reduced while aromatic silyl ethers, far less reactive, are preserved. Adapted from ref. 91.

In a similar vein, Brook *et al.* ^91,92^ suggested the conversion of lignin into aryl silyl ethers through concomitant reductive degradation and silylation with various hydrosilanes (Scheme 12). The authors determined the relative reactivity of each functional group present in lignin by sequentially adding molar equivalents of pentamethyldisiloxane.^91^ Drawing their inspiration from the Piers–Rubinsztajn reaction, which can be used to access silicone elastomer networks by cross linking hydrosilicones and alkoxysilanes, *e.g.* TEOS, the authors also prepared reinforced silicone elastomers by replacing TEOS with silylated lignin obtained *in situ*.^92^ Complete desilylation was also demonstrated using tetra-*n*-butylammonium fluoride.^91^ Thus, this approach can be seen as a versatile tool for selective tandem cleavage-functionalization of lignin.

**Scheme 13:**
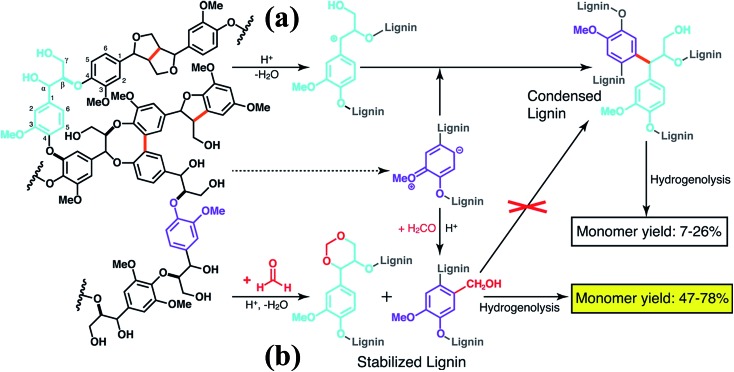
Lignin monomer production during bimass depolymerization. (a) Conventional lignin extraction in acidic conditions. Lignin ether bonds are cleaved while stable carbon–carbon bonds are formed. (b) Lignin extraction in the presence of formaldehyde, which hinders the formation of carbon–carbon bonds through electrophilic aromatic substitution (hydroxyalkylation) and reaction with 1,3-diol units of β-*O*-4 linkages to yield 1,3-dioxane units. Reproduced with permission from ref. 93. All rights reserved. Copyright 2016, American Association for the Advancement of Science.

More recently, Luterbacher *et al.* ^93^ reported on the use of formaldehyde to suppress irreversible condensations by interunit C–C coupling, which typically occur during biomass delignification, and promote lignin monomer production *via* hydrogenolysis (Scheme 13). Unlike previous examples that focused on the use of functional lignin and lignin residues for specific applications, this work exploited the chemical modification of lignin as a way to facilitate its deconstruction. In particular, formaldehyde was shown to form stable six-membered 1,3-dioxane rings with the diol systems present on the lignin side chains as well as to introduce hydroxymethyl groups on the aromatic backbone, thereby blocking most of the sites available to C–C formation. The monomer yield was significantly increased with respect to conventional strategies (Scheme 13) and the process is regarded as sustainable considering that formaldehyde is relatively inexpensive and could be produced from biomass. Furthermore, the authors showed that residual unreacted formaldehyde could be recovered quantitatively from the reaction mixture.^93^

Considering the recent proliferation of methods for the chemical deconstruction of lignin,^6,94^ functionalization strategies should not be restricted to polymeric lignins but should also deal with the valorization of mono aromatic compounds that can be produced from it. Up to now, a limited number of studies reported on the selective production and functionalization of low-molecular-weight aromatic compounds obtained from lignin either through chemical^95,96^ or enzymatic^97^ processes. Schmidt *et al.* described recently the preparation of a non-ionic surfactant by functionalizing lignin oligomers obtained by catalytic hydrogenolysis with poly(ethylene oxide).^96^ The resulting product was efficiently used as stabilizer for the emulsion polymerization of styrene, showcasing the possibility of valorizing lignin into an interesting commodity chemical. The group of Westwood, developed a method for the deconstruction of birch wood lignin based on a chemoselective oxidation of β-*O*-4 linkages promoted by DDQ/*t*BuONO/O_2_, followed by reaction with zinc. The protocol is characterized by a good selectivity for the production of a particular sinapyl based building block. The potential of such compound was thus assessed by several chemical transformations aimed at the preparation of polymerizable monomers and beta amino acid precursors among others.^95^ Ohta *et al.* ^97^ proposed the enzymatic cascade synthesis of phenylpropanone platform monomers followed by their chemical conversion into various derivatives. Even though the authors questioned the cost-effectiveness of their approach, they proposed to combine the synthesis of phenylpropanone platform monomers with the conversion of the remaining lignin fraction into functional materials. Such integrative approach that promotes the full and interrelated valorization of lignin into a portfolio of value added products is presumably the direction to follow in the next years.

## 5. Expanding the sustainable chemical space: alternative feedstocks and processing strategies

**Scheme 14:**
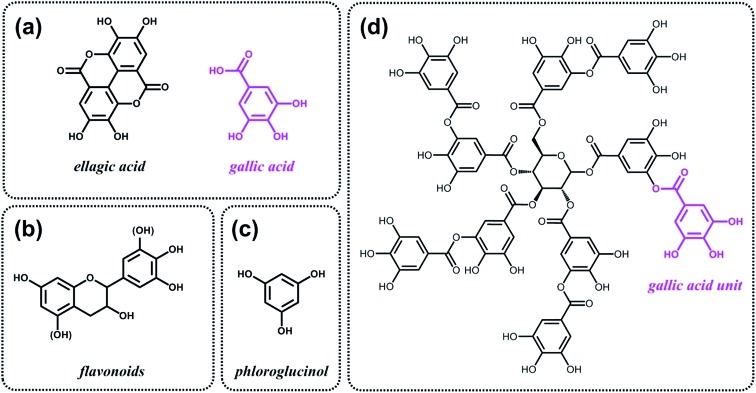
Typical monomers found in (a) hydrolysable tannins, (b) condensed tannins and (c) phlorotannins. (d) Structure of a typical hydrolysable tannin made of gallic acid monomers. This tannin is commercial and also known as tannic acid.

As discussed above, the expansion of the chemical space accessible to biorefinery consists mainly in the production of secondary building blocks through the covalent modification of those platform chemicals that can be obtained *via* biomass deconstruction. So far, examples related to the valorization of lignocellulosic molecules have been discussed. However, despite the great diversity of molecules, the chemical space accessible from woody biomass is still limited. One of the great challenges for biorefinery will be therefore the search for alternative renewable feedstocks that upon valorization can complement the chemical space associated to lignocellulose. Besides lignins, for example, other natural polyphenols have been suggested as starting material for the preparation of functional macromolecules and materials. Amongst them, tannins are particularly attractive as they are the most abundant source of natural aromatic biomolecules after lignins.^98^ Tannins can be found both in vascular – as hydrolysable and condensed tannins (Scheme 14a and b) – and non-vascular plants such as brown algae – as phlorotannins (Scheme 14c). Interestingly, they display a less complex structure than lignin – with molar masses between 500 and 3000 g mol^–1^ – and are soluble in water and few other polar solvents. Tannins are mostly constituted of phenols interlinked through ester, ether or carbon–carbon bonds. The constituting building blocks feature alternative substitution patterns compared to lignin phenols. However, they can undergo most of the reactions reported earlier for lignins, which involve hydroxyl groups or electrophilic aromatic substitutions. Like lignin, tannin displays heterogeneous reactivity depending on its source (vascular *versus* non-vascular), class (hydrolysable *versus* condensed) and even structure (resorcinol *versus* phloroglucinol rings in condensed tannins; Scheme 14b). While a few recent examples will be discussed in the following, we invite the readers to refer to different works for a comprehensive overview regarding chemical modification of tannins.^98^

**Scheme 15:**
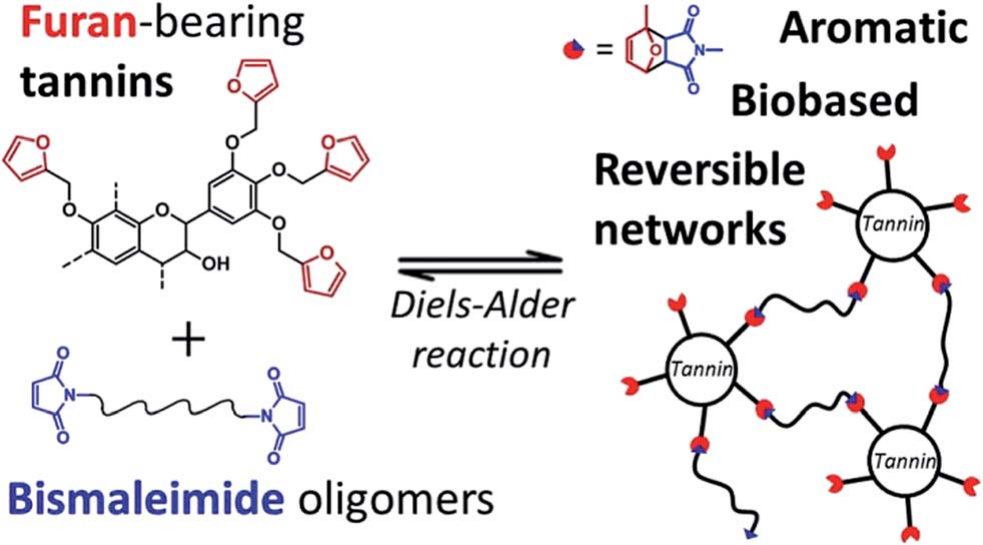
Thermo-reversible polymer networks prepared from furan-bearing condensed tannins and bismaleimide linkers. Tannin was functionalized through ring-opening reactions with furfuryl glycidyl ether. Reproduced with permission from ref. 100. All rights reserved. Copyright 2017, American Chemical Society.

Recent studies have highlighted the great potential of tannins to synthesize aromatic polymer networks. For instance, Caillol *et al.* ^99^ reported on the epoxidation of green tea tannins – a complex mixture of flavonoids and gallic acid – with epichlorohydrin to yield glycidyl ether pre-polymers. Such compounds were used for the formulation of bio-based aromatic epoxy resins with high crosslinking density. More recently, Avérous *et al.* ^100^ designed thermoreversible polymer networks from furan-bearing condensed tannins and bismaleimide linkers (Scheme 15). The transition from gel to liquid was observed upon heating at 120 °C. The reversibility of the cross-linking was tuned through Diels–Alder and retro Diels–Alder reactions (Scheme 15). The authors anticipated a broad range of applications for this class of compounds, going from reversible adhesives to self-healing materials.

Like lignins, tannins display suitable structures, *i.e.* natural aromatic character and high carbon content, for the preparation of partially graphitized carbons. Celzard *et al.* ^101^ developed the synthesis of aminated aromatic hydrogels through the treatment of condensed tannin with ammonia and subsequent hydrothermal carbonization. After drying and pyrolysis, porous nitrogen-doped carbons were obtained and tested as electrode materials for supercapacitors. In a similar fashion, Titirici *et al.* ^102^ proposed to use phloroglucinol, the monomer unit of phlorotannins (Scheme 14c), together with d-glucosamine and/or *N*-acetyl-d-glucosamine for the synthesis of nitrogen-doped carbon aerogels. Such materials were tested as metal-free oxygen reduction electrocatalysts.

Besides aromatic polymer networks and heteroatom-doped carbons, tannins were also employed for the design of hybrid structures. Caruso *et al.* ^103,104^ described a strategy that takes advantage of coordination interactions between tannic acid (Scheme 14d) and metal ions to engineer coatings^103^ and functional gels.^104^ When group (IV) metal ions were used, in particular titanium(iv) bis(ammonium lactato)dihydroxide, metallogels were easily obtained.^104^ Such metallogels showed peculiar properties amongst which high adhesive strength, self-healing ability and injectability. The authors also demonstrated the possibility to incorporate various functional materials into the metallogel, such as magnetic nanoparticles, graphene oxide and carbon nanotubes, thereby offering a versatile platform for the design of composite materials.

Even though tannin offers interesting opportunities, its production remains limited as compared with lignin. While more than 50 million tons of lignin are extracted annually worldwide by the paper industry,^74^ annual production of tannin reaches only 0.2 million tons.^98^ Furthermore, the annual production of lignin might significantly increase in the near future, as lignin is a major by-product from the production of forestry ethanol.^95^ As a consequence, tannin should be used as precursor for the industrial production of those specialty chemicals or functional macromolecules and materials that are required in relatively small quantities.

Moving away from the realm of plants, alternative and complementary chemical spaces can be explored by diversifying the renewable feedstock. In this regard, biological waste produced by established commercial sectors will offer exceptional opportunities.^105^ In particular, the production of nitrogen-containing furan derivatives^106,107^ (*e.g.* 3-acetamido-5-acetylfuran), nitrogen-containing polyols^108^ and heterocyclic compounds^109^ (*e.g.* pyrrole) were reported during fractionation of crustacean shells.^110,111^ Nitrogen-doped porous carbons were also obtained either from chitin/chitosan monomers, as mentioned earlier,^102^ or directly from lobster shells.^112^ In a similar way, chitin based films^113^ and fibers^114^ are becoming available through novel processing strategies involving ionic liquids. Shell biorefinery, as defined by Yan *et al.*,^110^ is still at an early stage of development but might open up new horizons and allow synthesizing new platform chemicals and functional materials that would be more difficult to obtain from woody biorefinery.

Relying on alternative renewable feedstock is however not the only possibility to explore new regions within the sustainable chemical space. While we have been referring primarily to chemical strategies for the deconstruction of lignocellulosic biomass and alternative feedstocks, it is important to recall the very important contributions coming from the area of microbial and biotechnological production of platform chemicals.^115,116^ In this regard, the example of itaconic acid IA represents a very interesting case study. IA is produced using biotechnological schemes, namely fungal fermentation processes of xylose or glucose.^117,118^ In particular, IA is produced at large scale *via* fermentation with *Aspergillus terreus*. Due to its pattern of chemical functionalities, which counts two carboxylic acid sites and an α,β-unsaturated double bond, IA offers large possibilities for derivatization and the synthesis of high value added compounds. IA has large potential in polymer chemistry, *i.e.* for polycondensation reactions, but it can also undergo radical polymerization involving the C=C bond. In organic fine chemistry, IA has large potential in the area of heterocycle synthesis.^119^ IA appears as a highly versatile and flexible starting material to access diverse five- and six-membered heterocycles (succinimides, pyrrolidones), but also heterocycles containing more than one heteroatom such as pyrazolidones. On the other side, itaconic anhydride is an interesting intermediate and allows to access 1,2,4 triazoles *via* a reaction with hydrazonamides. These compounds are potentially useful for their antimicrobial activities.^120^ More recently, Sperry *et al.* reported the synthesis of a series of biomass derived N-heterocycles including indoles, dihydroindolizinones and carbazoles. All these compounds are obtained from dimethyl itaconate and pyrrole.^121^ Interestingly, pyrrole can also be prepared on the basis of renewable precursors, showcasing the synthetic potential that exists within the sustainable chemical space.

IA and its derivatives allow also the access to oxygen and sulfur containing heterocycles. As an example, aldol condensation of diethyl itaconate with various ketones can be used to afford lactones. As already reported in the mid-1970s, this methodology can be applied to the synthesis of natural products such as (±)-nephrosterininc acid and (±)-protolichesterinic acid, two important members of the family of paraconic acids.^122^ These compounds are known for their biological activities in particular for their antibacterial, antifungal, antitumor and growth-regulating effects.^123^ In summary, IA and its derivatives appear as highly versatile building blocks to access broad libraries of architecturally diverse heterocycles following semisynthetic approaches. While IA is obtained by biotechnological means, chemical strategies can be used to access a variety of N, O and S substituted heterocycles.

## 6. Conclusions

Biorefinery, *i.e.* the valorization of the large variety of complex biopolymers within biomass, provides nowadays access to a diverse pool of primary building blocks, such as levulinic acid, furans and polyphenols. These compounds feature a number of chemical functionalities that can be exploited for the synthesis of high value chemicals and materials. However, for many of these applications, such building blocks cannot be utilized “as synthesized”, and further covalent modifications or functionalization are necessary. Particularly relevant are those synthetic approaches that involve the combination of biomass building blocks with additional sustainable chemicals. Such transformations expand the accessible biomass-derived chemical space providing secondary building blocks that figure as component of bio-active molecules or advanced materials. In this perspective, we reported a selection of recent examples dealing with the synthesis of secondary biomass based building blocks. All these examples show the high versatility of bio-sourced platform chemicals for the preparation of fine chemicals and advanced materials. In particular, novel methods for the transformation of carbohydrate derived primary building blocks like levulinic acid, γ-valerolactone and furfurals into bioactive molecules have been presented. Interestingly, highly complex compounds including optically active molecules were also reported. New chemical strategies for the synthesis of bio-based substituted benzenes derived from furans *via* Diels–Alder chemistry were also described, highlighting the potential of this approach to impact the preparation of established commodities like PET. Moreover, examples describing the functionalization and chemical modification of lignin, one of the most important renewable aromatic feedstock, have also been included, showcasing several strategies to expand the sustainable aromatic chemical space. Besides covalent modifications of lignocellulosic biomass derived synthons, alternative chemicals covering a complementary chemical space can be directly obtained by diversifying the renewable feedstocks. In this regard, possibilities related to the valorization of tannins or wastes like sea shells have been commented. All in all, the valorization of bio-sourced molecules into fine chemicals is still in its infancy. In the future, however, numerous opportunities tailored on the availability of different renewable feedstocks can be expected to expand the existing sustainable chemical space.

## References

[cit1] Schellnhuber H. J., Rahmstorf S., Winkelmann R. (2016). Nat. Clim. Change.

[cit2] IEA, IEA bioenergy Task 42 on biorefineries: co-production of fuels, chemicals, power and materials from biomass, in Minutes of the third Task meeting, Copenhagen, Denmark, 25–26 March 2007, http://www.biorefinery.nl/ieabioenergy-task42/, 2008.

[cit3] Cherubini F. (2010). Energy Convers. Manage..

[cit4] Liu J., Mooney H., Hull V., Davis S. J., Gaskell J., Hertel T., Lubchenco J., Seto K. C., Gleick P., Kremen C., Li S. (2015). Science.

[cit5] Armaroli N., Balzani V. (2016). Chem.–Eur. J..

[cit6] Graglia M., Kanna N., Esposito D. (2015). ChemBioEng Rev..

[cit7] Dusselier M., Mascal M. and Sels B. F., in Selective Catalysis for Renewable Feedstocks and Chemicals, ed. K. M. Nicholas, Springer International Publishing, Cham, 2014, pp. 1–40, 10.1007/128_2014_544.

[cit8] Esposito D., Antonietti M. (2015). Chem. Soc. Rev..

[cit9] Besson M., Gallezot P., Pinel C. (2014). Chem. Rev..

[cit10] Gilkey M. J., Xu B. (2016). ACS Catal..

[cit11] Rinaldi R., Jastrzebski R., Clough M. T., Ralph J., Kennema M., Bruijnincx P. C. A., Weckhuysen B. M. (2016). Angew. Chem., Int. Ed..

[cit12] Climent M. J., Corma A., Iborra S. (2014). Green Chem..

[cit13] Ruppert A. M., Weinberg K., Palkovits R. (2012). Angew. Chem., Int. Ed..

[cit14] Kirkpatrick P., Ellis C. (2004). Nature.

[cit15] Van Wouwe P., Dusselier M., Vanleeuw E., Sels B. (2016). ChemSusChem.

[cit16] Shen X., Shamshina J. L., Berton P., Gurau G., Rogers R. D. (2016). Green Chem..

[cit17] Kaushik M., Moores A. (2016). Green Chem..

[cit18] Sherwood J., De bruyn M., Constantinou A., Moity L., McElroy C. R., Farmer T. J., Duncan T., Raverty W., Hunt A. J., Clark J. H. (2014). Chem. Commun..

[cit19] Molinari V., Antonietti M., Esposito D. (2014). Catal. Sci. Technol..

[cit20] Shuai L., Luterbacher J. (2016). ChemSusChem.

[cit21] Heinze T. and Liebert T., in Polymer Science: A Comprehensive Reference, ed. M. Möller, Elsevier, Amsterdam, 2012, pp. 83–152, 10.1016/B978-0-444-53349-4.00255-7.

[cit22] Habibi Y. (2014). Chem. Soc. Rev..

[cit23] Stubba D., Lahm G., Geffe M., Runyon J. W., Arduengo A. J., Opatz T. (2015). Angew. Chem., Int. Ed..

[cit24] Pileidis F. D., Titirici M.-M. (2016). ChemSusChem.

[cit25] Chieffi G., Braun M., Esposito D. (2015). ChemSusChem.

[cit26] Ortiz-Cervantes C., Flores-Alamo M., Garcia J. J. (2016). Tetrahedron Lett..

[cit27] Touchy A. S., Siddiki S., Kon K., Shimizu K. (2014). ACS Catal..

[cit28] Ledoux A., Kuigwa L. S., Framery E., Andrioletti B. (2015). Green Chem..

[cit29] Wei Y., Wang C., Jiang X., Xue D., Liu Z.-T., Xiao J. (2014). Green Chem..

[cit30] Vidal J. D., Climent M. J., Concepcion P., Corma A., Iborra S., Sabater M. J. (2015). ACS Catal..

[cit31] Li Z., Tang X., Jiang Y., Zuo M., Wang Y., Chen W., Zeng X., Sun Y., Lin L. (2016). Green Chem..

[cit32] Swanston J., in Ullmann's Encyclopedia of Industrial Chemistry, Wiley-VCH Verlag GmbH & Co. KGaA, 2000, 10.1002/14356007.a26_793.pub2.

[cit33] Anderson R. J., Compton B. J., Tang C. W., Authier-Hall A., Hayman C. M., Swinerd G. W., Kowalczyk R., Harris P., Brimble M. A., Larsen D. S., Gasser O., Weinkove R., Hermans I. F., Painter G. F. (2015). Chem. Sci..

[cit34] Joshi S. D., Kumar D., Dixit S. R., Tigadi N., More U. A., Lherbet C., Aminabhavi T. M., Yang K. S. (2016). Eur. J. Med. Chem..

[cit35] Alonso D. M., Wettstein S. G., Dumesic J. A. (2013). Green Chem..

[cit36] Gu Y., Jerome F. (2013). Chem. Soc. Rev..

[cit37] Diaz-Rodriguez A., Borzecka W., Lavandera I., Gotor V. (2014). ACS Catal..

[cit38] Datrika R., Kallam S. R., Khobare S. R., Gajare V. S., Kommi M., Mohan H. R., Vidavalur S., Pratap T. V. (2016). Tetrahedron: Asymmetry.

[cit39] Tukacs J. M., Fridrich B., Dibo G., Szekely E., Mika L. T. (2015). Green Chem..

[cit40] Vobecka Z., Wei C., Tauer K., Esposito D. (2015). Polymer.

[cit41] Wei C., Esposito D., Tauer K. (2016). Polym. Degrad. Stab..

[cit42] Bohre A., Dutta S., Saha B., Abu-Omar M. M. (2015). ACS Sustainable Chem. Eng..

[cit43] Chatterjee M., Ishizaka T., Kawanami H. (2016). Green Chem..

[cit44] Roylance J. J., Choi K. S. (2016). Green Chem..

[cit45] Ryabukhin D. S., Zakusilo D. N., Kompanets M. O., Tarakanov A. A., Boyarskaya I. A., Artamonova T. O., Khohodorkovskiy M. A., Opeida I. O., Vasilyev A. V. (2016). Beilstein J. Org. Chem..

[cit46] Villain-Guillot P., Gualtieri M., Bastide L., Roquet F., Martinez J., Amblard M., Pugniere M., Leonetti J. P. (2007). J. Med. Chem..

[cit47] Bottari G., Kumalaputri A. J., Krawczyk K. K., Feringa B. L., Heeres H. J., Barta K. (2015). Chemsuschem.

[cit48] Chieffi G., Giordano C., Antonietti M., Esposito D. (2014). J. Mater. Chem. A.

[cit49] Yan T., Feringa B. L., Barta K. (2016). ACS Catal..

[cit50] Koh P. F., Loh T. P. (2015). Green Chem..

[cit51] Sowmiah S., Veiros L. F., Esperanca J., Rebelo L. P. N., Afonso C. A. M. (2015). Org. Lett..

[cit52] Villard R., Robert F., Blank I., Bernardinelli G., Soldo T., Hofmann T. (2003). J. Agric. Food Chem..

[cit53] Kirchhecker S., Troger-Muller S., Bake S., Antonietti M., Taubert A., Esposito D. (2015). Green Chem..

[cit54] Diels O., Alder K. (1929). Ber. Dtsch. Chem. Ges. A.

[cit55] Lanzafame P., Centi G., Perathoner S. (2014). Chem. Soc. Rev..

[cit56] Bruijnincx P. C. A., Weckhuysen B. M. (2013). Angew. Chem., Int. Ed..

[cit57] Zhang M., Yu Y. (2013). Ind. Eng. Chem. Res..

[cit58] Williams C. L., Chang C.-C., Do P., Nikbin N., Caratzoulas S., Vlachos D. G., Lobo R. F., Fan W., Dauenhauer P. J. (2012). ACS Catal..

[cit59] Kim T.-W., Kim S.-Y., Kim J.-C., Kim Y., Ryoo R., Kim C.-U. (2016). Appl. Catal., B.

[cit60] Wijaya Y. P., Suh D. J., Jae J. (2015). Catal. Commun..

[cit61] Wijaya Y. P., Kristianto I., Lee H., Jae J. (2016). Fuel.

[cit62] Green S. K., Patet R. E., Nikbin N., Williams C. L., Chang C.-C., Yu J., Gorte R. J., Caratzoulas S., Fan W., Vlachos D. G., Dauenhauer P. J. (2016). Appl. Catal., B.

[cit63] Pacheco J. J., Davis M. E. (2014). Proc. Natl. Acad. Sci. U. S. A..

[cit64] Pacheco J. J., Labinger J. A., Sessions A. L., Davis M. E. (2015). ACS Catal..

[cit65] Pang J., Zheng M., Sun R., Wang A., Wang X., Zhang T. (2016). Green Chem..

[cit66] Luciani-Torres M. G., Moore D. H., Goodson W. H., Dairkee S. H. (2015). Carcinogenesis.

[cit67] Gandini A., Lacerda T. M., Carvalho A. J. F., Trovatti E. (2016). Chem. Rev..

[cit68] Burgess S. K., Kriegel R. M., Koros W. J. (2015). Macromolecules.

[cit69] https://www.avantium.com/yxy/yxy-technology/#process .

[cit70] Mahmoud E., Yu J., Gorte R. J., Lobo R. F. (2015). ACS Catal..

[cit71] Beerthuis R., Rothenberg G., Shiju N. R. (2015). Green Chem..

[cit72] Thiyagarajan S., Genuino H. C., van der Waal J. C., de Jong E., Weckhuysen B. M., van Haveren J., Bruijnincx P. C. A., van Es D. S. (2016). Angew. Chem., Int. Ed. Engl..

[cit73] Pehere A. D., Xu S., Thompson S. K., Hillmyer M. A., Hoye T. R. (2016). Org. Lett..

[cit74] Kai D., Tan M. J., Chee P. L., Chua Y. K., Yap Y. L., Loh X. J. (2016). Green Chem..

[cit75] Auvergne R., Caillol S., David G., Boutevin B., Pascault J.-P. (2014). Chem. Rev..

[cit76] Upton B. M., Kasko A. M. (2016). Chem. Rev..

[cit77] Tao J., Hosseinaei O., Delbeck L., Kim P., Harper D. P., Bozell J. J., Rials T. G., Labbe N. (2016). RSC Adv..

[cit78] Qian Y., Zhang Q., Qiu X. Q., Zhu S. P. (2014). Green Chem..

[cit79] Liu H. L., Chung H. Y. (2016). Macromolecules.

[cit80] Over L. C., Meier M. A. R. (2016). Green Chem..

[cit81] Duval A., Lange H., Lawoko M., Crestini C. (2015). Biomacromolecules.

[cit82] Nilsson T. Y., Wagner M., Inganas O. (2015). Chemsuschem.

[cit83] Liu X. H., Yin H., Zhang Z. X., Diao B. S., Li J. (2015). Colloids Surf., B.

[cit84] Kai D., Low Z. W., Liow S. S., Karim A. A., Ye H. Y., Jin G. R., Li K., Loh X. J. (2015). ACS Sustainable Chem. Eng..

[cit85] Deng Y. H., Zhao H. J., Qian Y., Lu L., Wang B. B., Qiu X. Q. (2016). Ind. Crops Prod..

[cit86] Zhao H. J., Wang Q. J., Deng Y. H., Shi Q., Qian Y., Wang B. B., Lu L., Qiu X. Q. (2016). RSC Adv..

[cit87] Graglia M., Pampel J., Hantke T., Fellinger T. P., Esposito D. (2016). ACS Nano.

[cit88] Budarin V. L., Clark J. H., Henschen J., Farmer T. J., Macquarrie D. J., Mascal M., Nagaraja G. K., Petchey T. H. M. (2015). Chemsuschem.

[cit89] Li Z. L., Xiao D., Ge Y. Y., Koehler S. (2015). ACS Appl. Mater. Interfaces.

[cit90] Mu L. W., Shi Y. J., Guo X. J., Wu J., Ji T., Chen L., Feng X., Lu X. H., Hua J., Zhu J. H. (2016). ACS Sustainable Chem. Eng..

[cit91] Zhang J. F., Chen Y., Brook M. A. (2014). ACS Sustainable Chem. Eng..

[cit92] Zhang J., Chen Y., Sewell P., Brook M. A. (2015). Green Chem..

[cit93] Shuai L., Amiri M. T., Questell-Santiago Y. M., Heroguel F., Li Y. D., Kim H., Meilan R., Chapple C., Ralph J., Luterbacher J. S. (2016). Science.

[cit94] Xu C., Arancon R. A. D., Labidi J., Luque R. (2014). Chem. Soc. Rev..

[cit95] Lancefield C. S., Ojo O. S., Tran F., Westwood N. J. (2015). Angew. Chem., Int. Ed..

[cit96] Schmidt B. V. K. J., Molinari V., Esposito D., Tauer K., Antonietti M. (2017). Polymer.

[cit97] Ohta Y., Hasegawa R., Kurosawa K., Maeda A. H., Koizumi T., Nishimura H., Okada H., Qu C., Saito K., Watanabe T., Hatada Y. (2017). ChemSusChem.

[cit98] Arbenz A., Averous L. (2015). Green Chem..

[cit99] Benyahya S., Aouf C., Caillol S., Boutevin B., Pascault J. P., Fulcrand H. (2014). Ind. Crops Prod..

[cit100] Duval A., Couture G., Caillol S., Avérous L. (2017). ACS Sustainable Chem. Eng..

[cit101] Braghiroli F. L., Fierro V., Szczurek A., Stein N., Parmentier J., Celzard A. (2015). Carbon.

[cit102] Brun N., Wohlgemuth S. A., Osiceanu P., Titirici M. M. (2013). Green Chem..

[cit103] Ejima H., Richardson J. J., Liang K., Best J. P., van Koeverden M. P., Such G. K., Cui J. W., Caruso F. (2013). Science.

[cit104] Rahim M. A., Bjornmalm M., Suma T., Faria M., Ju Y., Kempe K., Mullner M., Ejima H., Stickland A. D., Caruso F. (2016). Angew. Chem., Int. Ed..

[cit105] Tuck C. O., Pérez E., Horváth I. T., Sheldon R. A., Poliakoff M. (2012). Science.

[cit106] Omari K. W., Dodot L., Kerton F. M. (2012). ChemSusChem.

[cit107] Chen X., Chew S. L., Kerton F. M., Yan N. (2014). Green Chem..

[cit108] Bobbink F. D., Zhang J., Pierson Y., Chen X., Yan N. (2015). Green Chem..

[cit109] Gao X., Chen X., Zhang J., Guo W., Jin F., Yan N. (2016). ACS Sustainable Chem. Eng..

[cit110] Chen X., Yang H., Yan N. (2016). Chem.–Eur. J..

[cit111] Yan N., Chen X. (2015). Nature.

[cit112] Preuss K., Kannuchamy V. K., Marinovic A., Isaacs M., Wilson K., Abrahams I., Titirici M.-M. (2016). J. Energy Chem..

[cit113] King C., Shamshina J. L., Gurau G., Berton P., Khan N. F. A. F., Rogers R. D. (2017). Green Chem..

[cit114] Barber P. S., Kelley S. P., Griggs C. S., Wallace S., Rogers R. D. (2014). Green Chem..

[cit115] Jang Y.-S., Kim B., Shin J. H., Choi Y. J., Choi S., Song C. W., Lee J., Park H. G., Lee S. Y. (2012). Biotechnol. Bioeng..

[cit116] Straathof A. J. J. (2014). Chem. Rev..

[cit117] Corma A., Iborra S., Velty A. (2007). Chem. Rev..

[cit118] Klement T., Buechs J. (2013). Bioresour. Technol..

[cit119] Medway A. M., Sperry J. (2014). Green Chem..

[cit120] Modzelewska-Banachiewicz B., Paprocka R., Mazur L., Saczewski J., Kutkowska J., Stepien D. K., Cyranski M. (2012). J. Mol. Struct..

[cit121] Bhusal R. P., Sperry J. (2016). Green Chem..

[cit122] Carlson R. M., Oyler A. R. (1975). Tetrahedron Lett..

[cit123] Hughes M. A., McFadden J. M., Townsend C. A. (2005). Bioorg. Med. Chem. Lett..

